# Flexible Five-in-One Microsensor for Real-Time Wireless Microscopic Diagnosis inside Electric Motorcycle Fuel Cell Stack Range Extender

**DOI:** 10.3390/mi12020103

**Published:** 2021-01-21

**Authors:** Chi-Yuan Lee, Chia-Hung Chen, Ti-Ju Lee, John-Shong Cheong, Yi-Cheng Liu, Yu-Chun Chen

**Affiliations:** 1 Yuan Ze Fuel Cell Center, Department of Mechanical Engineering, Yuan Ze University, Taoyuan 32003, Taiwan; royli0504@gmail.com (T.-J.L.); johnshong1018@gmail.com (J.-S.C.); pierce0922@gmail.com (Y.-C.L.); aa0989959498@gmail.com (Y.-C.C.); 2 HOMYTECH Global Co., Ltd., Taoyuan 33464, Taiwan; chenjahon@gmail.com

**Keywords:** electric motorcycle, PEMFC stack, range extender, flexible five-in-one microsensor, wireless microscopic diagnosis

## Abstract

The focus of research and development on electric motorcycle range extender are system integration and energy regulation and management but the present fuel cell stack range extender still has defects, such as large volume, heavy weight and high cost. Its volume and weight will have a strong impact on the endurance of electric motorcycle. The bipolar plate takes most volume and weight of a proton exchange membrane fuel cell (PEMFC) stack and it is the key component influencing the overall power density and cost. Therefore, how to thin and lighten the bipolar plate and to enhance the performance and life of PEMFC stack is an urgent research subject to be solved for the moment and will be the key to whether the PEMFC stack range extender can be put in the electric motorcycle or not. In addition, the internal temperature, humidity, flow, voltage and current in the operation of PEMFC stack will influence its performance and life and the overall performance and life of fuel cell stack will be directly influenced by different external operating conditions. As nonuniform distribution of temperature, humidity, flow, voltage and current will occur in various regions inside the fuel cell stack, this study will use micro-electro-mechanical systems (MEMS) technology to develop a flexible five-in-one microsensor, which is embedded in the PEMFC stack range extender for real-time wireless microscopic diagnosis and the reliability test is performed, so that the actual operating condition inside the fuel cell stack range extender can be mastered instantly and correctly and the internal information is fed back instantly, the fuel cell stack range extender control system can be modified to the optimum operating parameters immediately, so as to enhance the performance and prolong the lifetime effectively.

## 1. Introduction

At present, battery storage capacity is limited and the driving range is short after a charging. A possible solution to this is to install a range extender on battery electric vehicles to increase the driving range of battery electric vehicles. The electric vehicle range extender is an additional energy storage component installed on the battery electric vehicle for increasing the running kilometrage of the vehicle. The electric energy can be supplied when the battery power is exhausted. It can supplement electric energy to the battery of an electric vehicle instantly, protecting the battery against over discharge. The electric vehicle range extender can supplement electric energy to the electric vehicle battery in an optimum charging state instantly, preventing the heating induced by battery over discharge and overcharge and electrolyte boiling evaporation. The optimal operating state can thus be maintained continuously, so the battery service life can be increased greatly.

Among the constituent elements of a proton exchange membrane fuel cell (PEMFC) stack, the bipolar plate accounts for almost 88% of total weight of fuel cell stack [1], to reduce the weight, the bipolar plate is a good cut-in point. It is indicated that many international corporations or research institutions studied bipolar plate in succession. For example, (1) Treadstone Technologies developed composite metal bipolar plate; (2) Argonne National Laboratory developed low cost metal bipolar plate [2]; (3) Wilberforce et al. studied the influence of geometric design of bipolar plate on the performance of PEMFC [3]; (4) Madadi et al. coated different materials on a metal bipolar plate to improve the performance of PEMFC [4].

The performance of PEMFC is mutually influenced by many operating parameters, such as temperature, humidity, pressure and flow. Lee et al. [5] used micro-electro-mechanical systems (MEMS) technology to integrate micro temperature and humidity sensors to measure local values of PEMFC. The study found that the temperature difference between Membrane Electrode Assembly (MEA) and a bipolar plate was 5.7 °C. In order to eliminate local CO_2_ to prevent vehicles from emitting exhaust gas, Hoeflinger et al. [6] used a PEMFC range extender system to optimize the air flow rate and pressure data, so as to enhance the performance. Tang et al. [7] used 25 μm thick Type-T thin film thermocouple to measure the temperature difference, which was about 1.5 °C. Lee et al. [8] embedded a microsensor in the vanadium redox battery, the accuracy and sensitivity of micro temperature sensor were 0.5 °C and 2 × 10^−3^ °C respectively. Fergany [9] used a SSO (Salp Swarm Optimizer) to optimize the operating parameters of PEMFC.

In terms of PEMFC, the temperature, humidity, pressure and flow can influence the cell performance and life. When it is too dry, the internal reaction of MEA is incomplete. When the gas is too wet, the membrane material is unlikely to retain moisture. Under different operating conditions, if the PEMFC has severe water accumulation, the runner will be blocked temporarily, the voltage of cell drops instantaneously, the cell performance degrades and the cell performance cannot be predicted. In order to measure the temperature distribution at all points of MEA, Inman et al. [10] used phosphor thermometry to design an optical fiber temperature sensor and embedded the optical fiber temperature sensor between the bipolar plate and the gas diffusion layer. However, the invasive measurement mode not only increases the cost but also influences the cell performance [11,12,13].

Tan et al. [14] used LC wireless sensor to detect the temperature and humidity according to the change in capacitance of resonant circuit. Huang et al. [15] used wireless sensor data collector and wireless sensor nodes of different functions for monitoring experiment data. The system composed of wireless sensors, Cloud Computing and storage, enables the researchers to perform local monitoring on a local scale or to perform remote data monitoring through Cloud. Jafer et al. [16] developed a wireless sensor network module; the resistance and capacitance values were measured by resistance temperature detector (RTD) and capacitive pressure sensor, the signals were digitally processed by analog to digital convertor (ADC) and sent by RF module to the measurement instrument, the wireless sensor module successfully measured the internal temperature and pressure information of water pipe. Huang et al. [17] developed an RLC (resistance-inductance-capacitance) resonant circuit and used Polydimethylsiloxane (PDMS) as the substrate of strain sensor. This circuit will induce coil, when the coil inductance changes, the value is derived from an equation.

At present, the bipolar plate of a PEMFC stack is still made of metal and graphite, because the metal bipolar plate has a smaller volume, lower cost and high mechanical strength. But it has lower electrical conductivity and resistance to galvanic corrosion, so the metal bipolar plate is inapplicable to the operating conditions in a harsh environment. The graphite has quite good chemical stability, corrosion resistance and electrical conductivity. The bipolar plate isolates the oxidation and reduction between two poles and collects current and it shall contact MEA equally to collect current effectively, so its material shall have quite good chemical stability, corrosion resistance and electrical conductivity. If the resistivity is too high, the internal resistance of battery increases greatly, the efficiency decreases, the electric energy is lost and the waste heat is accumulated. Therefore, there must be good electrical conductivity and low contact resistance [18,19,20]. To sum up, the operating environment shall be considered in selecting the material of bipolar plate. The graphite is suitable for harsh environment, whereas the metal bipolar plate is suitable for general conditions.

## 2. Design and Production of PEMFC Stack

**Figure 1:**
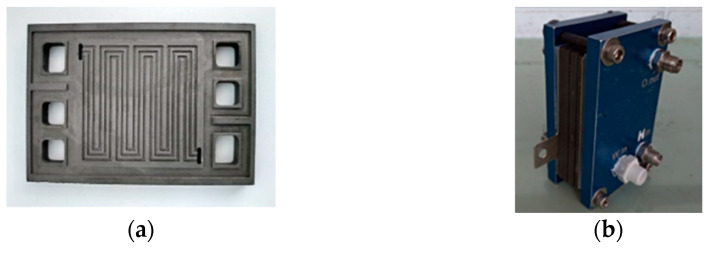
(**a**) Stereogram of runner plate; (**b**) fuel cell stack.

This study has successfully developed the PEMFC stack, the bipolar plate runner design uses serpentine flow field as shown in Figure 1a. The assembled entity is shown in Figure 1b. The bipolar plate specifications as shown in Table 1. The serpentine flow field is one of the most frequently used forms used in fuel cell stack runner plate design. This design form results in faster fuel inlet transmission speed, so the fuel in the front edge of runner is likely to be conveyed via the diffusion layer to the catalyst layer, better performance distribution can be obtained.

## 3. Integrated Design and Sensing Principle of Microsensor

This study developed a special flexible five-in-one microsensor, which could be embedded in the fuel cell stack range extender of electric motorcycle. The micro temperature sensor, micro humidity sensor, micro flow sensor, micro voltage sensor and micro current sensor were integrated into the flexible five-in-one microsensor by using MEMS technology, which was corrected and the thermal shock and constant temperature and humidity tests and durability test for the flexible five-in-one microsensor were performed. Afterwards, the flexible five-in-one microsensor was embedded in the PEMFC stack, so as to simultaneously measure the local states of internal temperature, humidity, flow, voltage and current in the operation of fuel cell stack.

### 3.1. Integrated Design of Flexible Five-in-One Microsensor

**Figure 2:**
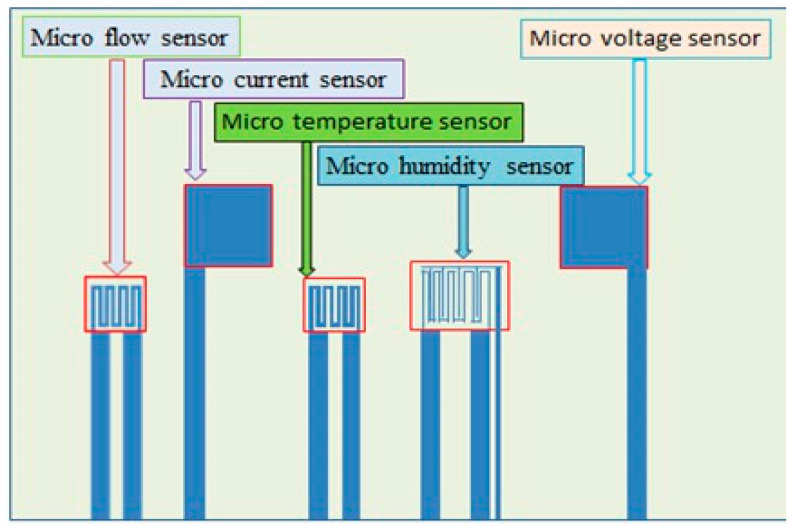
Schematic diagram of integration of flexible five-in-one microsensor.

The flexible five-in-one microsensor is developed by using MEMS technology, which can measure the temperature, humidity, flow, voltage and current simultaneously. Its sensing principle is described below. Figure 2 is the integrated design drawing of the flexible five-in-one (temperature, humidity, flow, voltage and current) microsensor designed in this study. The temperature sensing area is 240 μm × 240 μm, the humidity sensing area is 270 μm × 270 μm, the flow sensing area is 240 μm × 240 μm, the voltage sensing area is 350 μm × 350 μm and the current sensing area is 350 μm × 350 μm.

### 3.2. Sensing Principle of Micro Flow Sensor

This study used hot-wire micro flow sensor, the sensing end is an electrical resistance heater, the heater is given a constant voltage to generate a heat source. When the fluid passes by the heat source, the heat is carried away, so that the temperature of heater changes. Due to the material properties, the resistance changes when the temperature changes. According to Ohm’s law, in the case of constant voltage, the current changes when the resistance changes, the values are measured according to this principle. If the process is in ideal situation and completely matching heat transfer and heat convection, the power supplied by power supply can be equated with the heat carried away by the fluid. The flow can be converted into electric signal output by constant temperature circuit design. In other words, the hot-wire micro flow sensor is a microsensor designed using the positive correlation between thermal energy dissipation rate of hot wire and fluid flow.

## 4. Process Development of Flexible Five-in-One Microsensor

**Figure 3:**
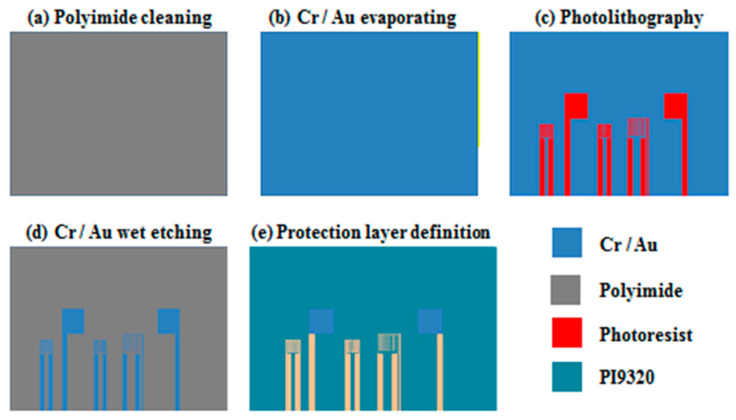
Process chart of flexible five-in-one microsensor.

**Figure 4:**
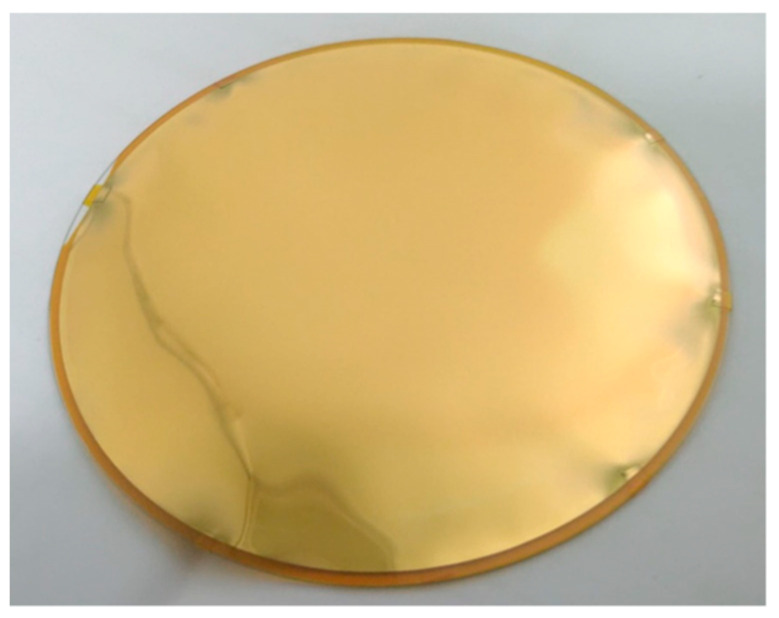
The polyimide (PI) substrate after chromium and gold film evaporation.

The production process of the flexible five-in-one microsensor is shown in Figure 3. (a) The PI substrate is cleaned with organic solutions acetone and methanol respectively and then the residual methanol, the surface dust and residual oil and fat are removed by DI water, so as to enhance the adhesive ability of thin film metal, 0.1 Å/s deposition rate for evaporation, (b) the Cr is evaporated by using E-beam evaporator as adhesion layer and the Au is evaporated as sensing layer is shown in Figure 4; (c) the pattern of micro temperature, humidity, flow, voltage and current sensor is defined by using photolithography process; (d) the pattern is transferred to the metal film of Cr and Au by wet etching; (e) the PI9320 is coated as protection layer and the voltage and current sensing areas and pins are defined by using photolithography process, the production of flexible five-in-one microsensor is completed.

## 5. Durability and Reliability Tests for Flexible Five-in-One Microsensor

### 5.1. Temperature Correction of Flexible Five-in-One Microsensor

**Figure 5:**
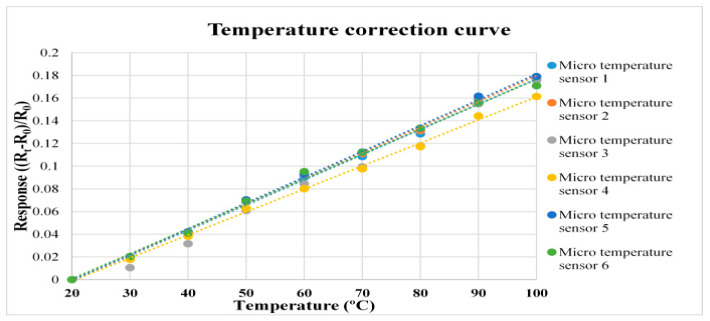
Correction curve of micro temperature sensor.

The flexible five-in-one microsensor and the thermometer of the BM-525 BRYMEN digital multimeter are put in DENG YNG DS45 Drying Oven (UNITED CORPS Co., Ltd., Lo San Village, Taiwan), the resistance value is extracted at intervals of 10 °C from 20 °C to 100 °C. The micro temperature sensor is corrected three times and the average value is taken, the measured correction curve shows that the micro temperature sensor has favorable linearity and reliability, as shown in Figure 5.

### 5.2. Humidity Correction of Flexible Five-in-One Microsensor

**Figure 6:**
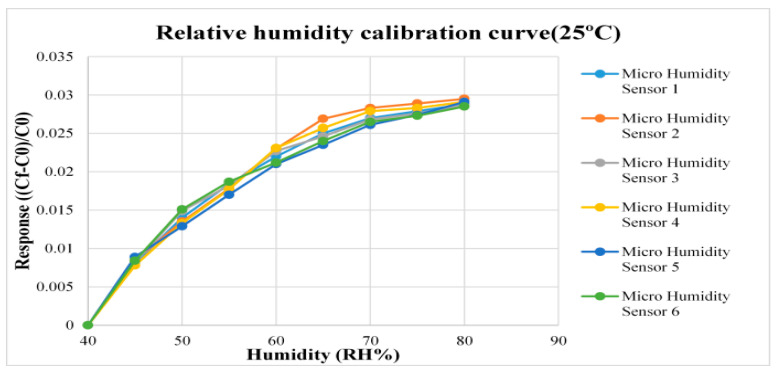
Correction curve of micro humidity sensor (25 °C).

**Figure 7:**
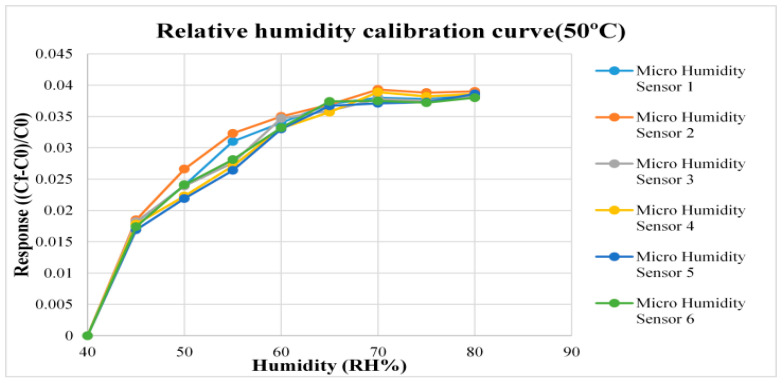
Correction curve of micro humidity sensor (50 °C).

For humidity correction, the constant temperature and humidity testing machine is used as environmental criteria, from relative humidity 40% to 80% and the temperatures 25 °C and 50 °C are used for correction, each time when the relative humidity recording point is increased, the heater of the micro humidity sensor is used for heating to completely evaporate the residual moisture at previous recording point, after 120 min stabilization, the NI PXI 2575 data acquisition unit is used to extract the capacitance value of micro humidity sensor instantly, so as to obtain the correction curve, as shown in Figure 6 and Figure 7.

## 6. Internal Measurement and Wireless Microscopic Diagnosis of Fuel Cell Stack

### 6.1. Comparison between Normal Flow Rate and Low Flow Rate and Operating Conditions of Wireless Sensor

In order to know the reaction inside the PEMFC stack in the operating environment of insufficient flow, the cell stack is supplied with hydrogen at a low flow rate (0.05 slpm), so as to observe the reaction of the cell stack in the operating environment at a low flow rate and to monitor the feasibility of the wireless sensor. The test conditions are shown in Table 2.

**Figure 8:**
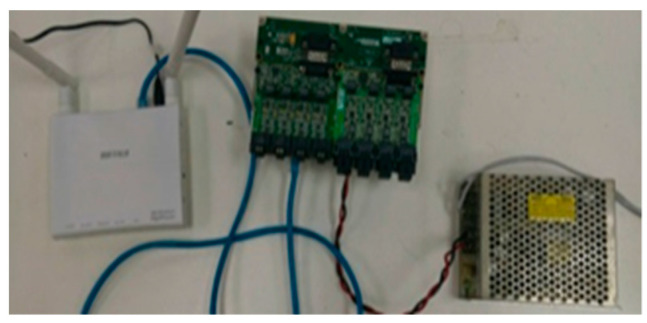
NI 9227 and NI 9219 wireless modules.

**Figure 9:**
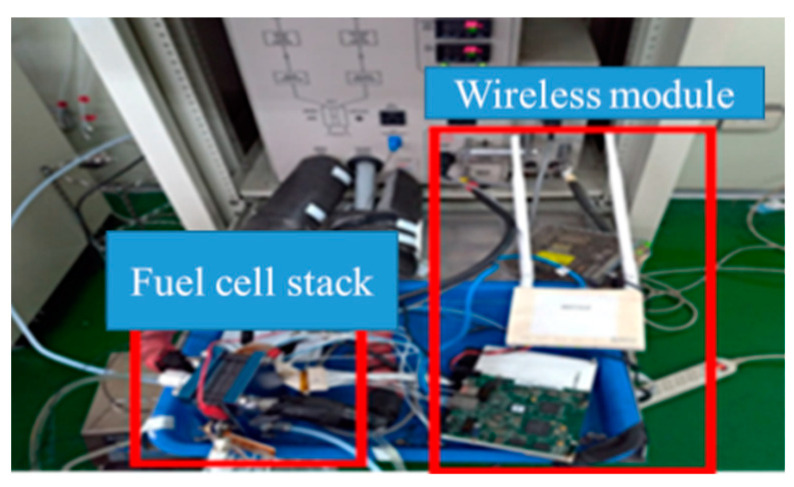
Wireless diagnosis system setup.

For the future application to electric motorcycles, this study must overcome the oversize of prior signal acquisition machine (NI PXI 2575), so the NI 9227 and NI 9219 wireless modules are further customized, as shown in Figure 8, so as to simplify the equipment for measuring signals and the measurement signals extracted by the data acquisition single plate are transmitted to the tablet PC display interface instantly coordinating with area data network, so that the flexible five-in-one microsensor is used for internal wireless remote diagnosis of fuel cell stack. The wireless system diagnosis setup of this study is shown in Figure 9.

#### 6.1.1. Fuel Cell Stack Performance Testing

**Figure 10:**
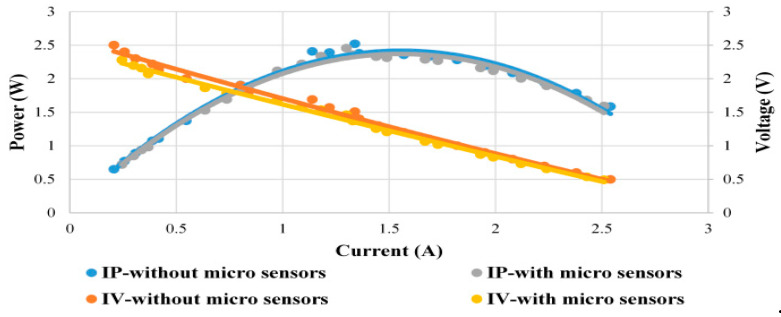
Performance comparison diagram with and without microsensor.

The performance curves of the fuel cell stacks with and without flexible five-in-one microsensor are compared in this study to test whether the flexible five-in-one microsensor has a strong impact on the performance of cell stack. As the flexible five-in-one microsensor developed in this study is very small, the total area of six flexible five-in-one microsensors is about 2.64% of the overall reaction area of MEA. The flexible five-in-one microsensor embedded in the cell stack influences about 2.58% of the performance of cell stack, as shown in Figure 10.

#### 6.1.2. Local Temperature Distribution Difference between Normal Flow Rate and Low Flow Rate

**Figure 11:**
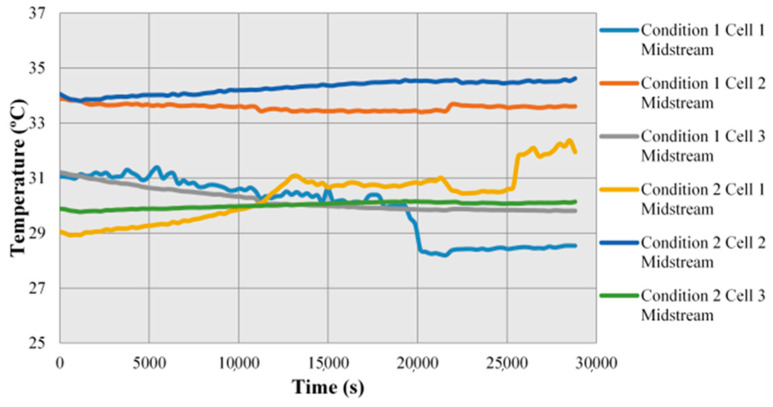
Local distribution of temperature in Condition 1 and Condition 2.

The fuel cell stack is supplied with normal flow (condition 1) and low flow (condition 2) for test in this study, the temperature change inside the fuel cell stack is observed. As shown in Figure 11, when the flow is sufficient, the internal temperature of cell stack drops in the early stage, because the flow is sufficient, there is adequate gas to carry the heat away from the cell stack. When the flow is low, the internal temperature of cell stack rises gradually, because there is no adequate gas inside to carry the heat away. It is also found in the figure that as the Cell 2 is in the middle of cell stack, the heat is likely to accumulate, the temperature is higher than Cell 1 and Cell 3.

#### 6.1.3. Local Relative Humidity Distribution Difference between Normal Flow Rate and Low Flow Rate

**Figure 12:**
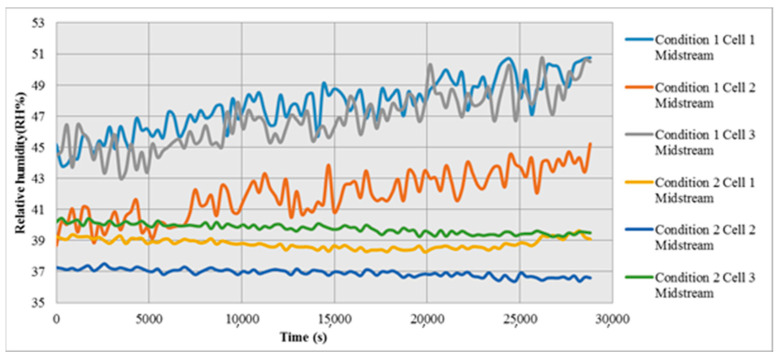
Local distribution of relative humidity in Condition 1 and Condition 2.

The relative humidity inside cell stack increases with time in condition 1, because there are more oxygen and hydrogen molecules when the fuel is sufficient, which can be combined to generate more water vapor. However, as the flow is insufficient in condition 2, there is less moisture, the relative humidity and humidity change are lower than that in condition 1, as shown in Figure 12.

#### 6.1.4. Local Flow Distribution Difference between Normal Flow Rate and Low Flow Rate

**Figure 13:**
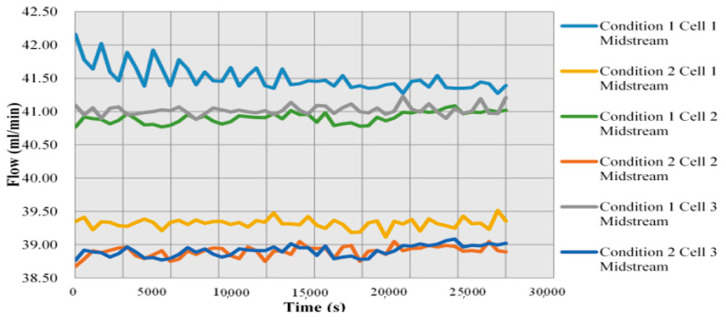
Local distribution of flow in Condition 1 and Condition 2.

As shown in Figure 13, when the flow is sufficient, the efficiency of cell stack increases with time and the gas consumption increases, so the flow decreases steadily with time. However, if the flow is insufficient, the gas distribution in the runner is very unstable and the snakelike runner design used in this study makes the flow more unstable.

**Figure 14:**
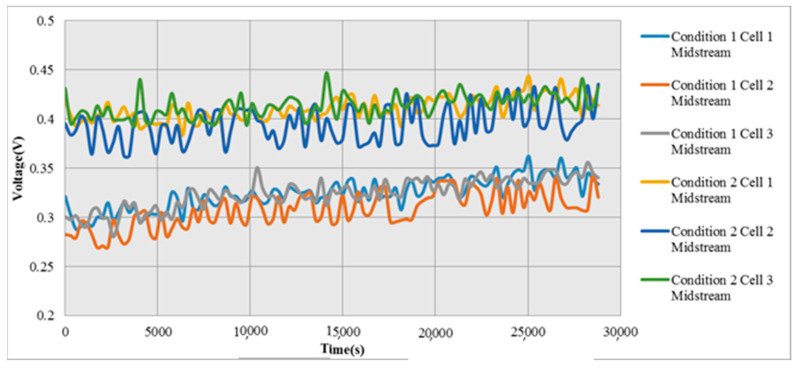
Local distribution of voltage in Condition 1 and Condition 2.

In condition 1, the voltage increases with time, because proton exchange membrane inside the cell stack is lost with time as the cell stack works. The corrosion of runner and the oxidation of collector plate induce the aging of cell stack. The aging of cell stack will increase the resistance inside cell stack, in order to maintain stable current output of cell stack, the voltage inside cell stack increases with time, as shown in Figure 14.

#### 6.1.5. Local Current Density Distribution Difference between Normal Flow Rate and Low Flow Rate

**Figure 15:**
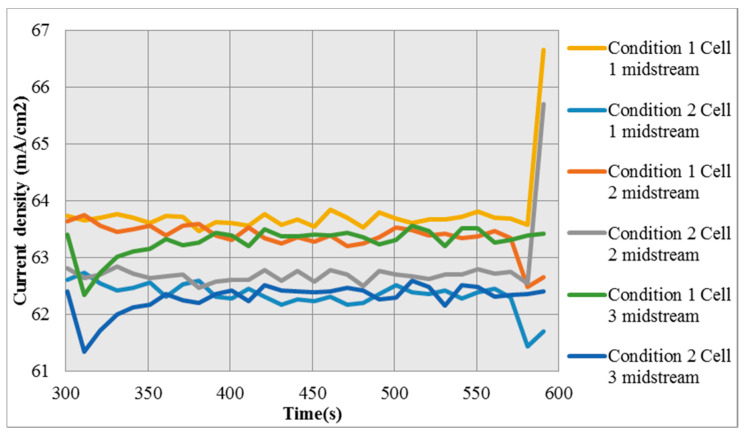
Local distribution of current density in Condition 1 and Condition 2.

In condition 1, the voltage of cell stack increases with time and the current density increases with time. The current density rises inside the cell stack but the loss inside the cell stack increases, the current density measured outside has not increased. There is higher voltage in condition 2, the current density is higher at the beginning but the current is not high as the flow is insufficient, so the current density in condition 2 is lower than that in condition 1, as shown in Figure 15.

## 7. Conclusions

The micro temperature, humidity, flow, voltage and current sensors are integrated on a 50 μm thick Polyimide (PI) foil substrate successfully by using MEMS technology in this study. This flexible five-in-one microsensor has five sensing functions, small thickness, small structural area, high sensitivity, real-time measurement and arbitrary placement. The flexible five-in-one microsensor can be embedded in the anode runner plate of cell stack without influencing the sealing condition of PEMFC stack. The local temperature, humidity, flow, voltage and current data inside the fuel cell stack are successfully extracted by NI PXI 2575 data acquisition unit and NI 9227 and NI 9219 wireless modules in the operational process of fuel cell stack.

## Figures and Tables

**Table 1:** Bipolar plate specifications.

Item	Condition
Bipolar plate thickness (mm)	6
Runner type	Anode, Cathode: Multiple (3 runners)
Runner depth (mm)	1
Runner width (mm)	1
Rib width (mm)	1

**Table 2:** Test conditions of high and low flow rates of fuel cell stack.

Item	Flow	Output Current	Temperature	Time
Condition 1	Hydrogen: 0.5 slpm Oxygen: 1.5 slpm	1 A	25 °C	8 h
Condition 2	Hydrogen: 0.05 slpm Oxygen: 1.5 slpm			
